# Nanocarriers for topical minoxidil in androgenetic alopecia: systematic review of preclinical and clinical evidence^☆^

**DOI:** 10.1016/j.abd.2026.501400

**Published:** 2026-07-03

**Authors:** Heloisa Da Rocha Picado Copesco, Gustavo Sartori Albertino, Filipe Rocha Lima, Antonio Claudio Tedesco, Marco Andrey Cipriani Frade

**Affiliations:** a Department of Internal Medicine, Division of Dermatology, Faculdade de Medicina de Ribeirão Preto, Hospital das Clínicas, Universidade de São Paulo, Ribeirão Preto, SP, Brazil; b Dermatology Division, Faculty of Medicine, Universidade de Ribeirão Preto, SP, Brazil; c Department of Biochemistry and Immunology, Faculdade de Medicina de Ribeirão Preto, Universidade de São Paulo, Ribeirão Preto, SP, Brazil; d Center of Nanotechnology and Tissue Engineering, Photobiology and Photomedicine Research Group, Faculdade de Filosofia, Ciências e Letras de Ribeirão Preto, Universidade de São Paulo, Ribeirão Preto, SP, Brazil

**Keywords:** Androgenetic alopecia, Drug delivery systems, Hair follicle, Minoxidil, Nanoparticles, Skin absorption

## Abstract

**Background:**

Androgenetic Alopecia (AGA) is the most prevalent hair disorder. Conventional topical minoxidil is limited by suboptimal follicular penetration, local adverse effects, and poor adherence; nanocarrier-based “nanominoxidil” systems may optimize delivery, but their clinical value remains uncertain.

**Objective:**

To synthesize experimental and clinical evidence on nanocarrier-based topical minoxidil for AGA.

**Methods:**

We systematically searched databases (2015–2024) for *in vitro*, *ex-vivo*, *in vivo*, and human studies evaluating nanocarriers < 1000 nm loaded with minoxidil versus conventional minoxidil, placebo, or no treatment. Primary outcomes were follicular and cutaneous penetration or retention; secondary outcomes included hair length, density and/or coverage, biomarker modulation, adverse events, and formulation stability. Risk of bias was assessed with SYRCLE and OHAT.

**Results:**

Of 410 records, 20 studies met eligibility criteria, predominantly preclinical, and one evaluated cutaneous tolerability in healthy volunteers. Across platforms (lipid, polymeric, hybrid and deformable systems, nanoemulsions), nanominoxidil consistently increased follicular deposition and cutaneous retention (≈2- to > 7-fold vs conventional solutions) and improved hair growth surrogates and angiogenic or stem-cell markers. Local tolerability was generally acceptable and systemic exposure was negligible when reported.

**Study limitations:**

Evidence is preclinical, with a single non-therapeutic human study; heterogeneity across models, comparators, doses, follow-up, and outcomes, and frequent lack of randomization, blinding, and standardized safety reporting, precluded meta-analysis and limited internal validity and generalizability.

**Conclusions:**

Nanotechnology-based minoxidil formulations enhance follicular targeting and hair-growth surrogate outcomes in experimental models and appear locally safe, but randomized trials in patients with AGA are required before nanominoxidil can be recommended for routine clinical use.

## Introduction

Androgenetic Alopecia (AGA), commonly known as male pattern baldness, is the most prevalent form of hair loss in both men and women. This condition results from the progressive miniaturization of hair follicles under genetic and androgenic influences, with dihydrotestosterone (DHT) playing a crucial role in this process.^1^

AGA affects approximately 30% of men under 30-years of age, increasing to approximately 50% after 50-years and up to 80% in individuals over 70-years.^2^ In women, the prevalence reaches approximately 10% in those over 50-years of age, potentially escalating to 40% among women in their 70 s.^3^ The pathophysiology of AGA involves shortening of the anagen phase of the hair cycle, an increase in the latency period known as the kenogen phase, and miniaturization of follicles, leading to finer and less pigmented hairs.^4^, ^5^ This condition currently has no definitive cure; however, therapeutic interventions aim to control its progression and improve hair aesthetics.

Minoxidil, originally a vasodilator used in the treatment of systemic arterial hypertension, is currently employed as both a topical and low-dose oral treatment (ranging from 0.5 to 5 mg/day) for AGA control.^6^ Its action involves metabolism to minoxidil sulfate, which opens ATP-dependent potassium channels, thereby promoting vasodilation in the scalp and stimulating hair growth.^7^ Minoxidil acts through multiple pathways (vasodilator, anti-inflammatory agent, inducer of the Wnt/β-catenin signaling pathway, and antiandrogen), and its exact mechanism of action is still not fully elucidated.^6^, ^7^, ^8^ This drug may also affect the hair cycle and influence the duration of the anagen and telogen phases.^8^ Despite its recognized efficacy and approval by agencies such as the Agência Nacional de Vigilância Sanitária (ANVISA) and the Food and Drug Administration (FDA),^9^ conventional topical minoxidil formulations face significant challenges, including low cutaneous penetration, localized irritation, and suboptimal treatment adherence due to the need for frequent application and cosmetic discomfort.^9^

To overcome these limitations, nanotechnology has emerged as a promising field in biomedicine and tissue engineering.^1^ Nanoparticles, which have been extensively explored, encompass various structures, such as micelles, liposomes, nanoemulsions, and polymeric and solid lipid nanoparticles (SLN), among others.^1^ The primary advantage of nanocarriers lies in their ability to optimize the active concentration, reduce the required dose, and achieve high therapeutic efficacy both *in vitro* and *in vivo* by effectively traversing biological barriers and optimizing drug transport. Parameters such as size (ideally between 1 and 100 nm for efficient barrier penetration), aqueous compatibility (often optimized by PEGylation to prolong systemic circulation and evade the reticuloendothelial system), surface charge, and geometry directly influence pharmacokinetics, pharmacodynamics, and cellular internalization.^1^ In dermatology, nanoparticles offer diverse possibilities for improving the targeted delivery of active compounds^2^ and serve as advanced drug delivery systems to optimize efficacy and safety. Despite these theoretical advantages, nanoformulations face several practical challenges, including stability issues, clearance by the reticuloendothelial system, biodistribution complexities, and considerations regarding industrial viability and therapeutic costs.^1^

In the context of AGA, the application of nanotechnology to minoxidil holds the potential to revolutionize its therapy, leading to ‘nanominoxidil’, in which nanocarriers such as liposomes, nanoemulsions, and solid lipid nanoparticles, are being developed to enhance the bioavailability of minoxidil, promote controlled release, improve follicular penetration, and mitigate potential adverse effects associated with oral medications, such as hypertrichosis and cardiac events.^10^, ^11^, ^12^

Nanobiotechnological minoxidil formulations are believed to offer superior efficacy and greater stability, with improved bioavailability compared with traditional minoxidil.^10^ However, despite the theoretical potential and promising evidence from isolated studies, a comprehensive and critical synthesis integrating preclinical and clinical data to evaluate the efficacy, safety, and translational applicability of these formulations is still lacking.^2^ Studies involving minoxidil nanoparticles are still scarce, and more clinical research is necessary to firmly establish their benefits and optimize their application in AGA therapy.^12^

We conducted a systematic review of the literature from the past 10-years to evaluate nanotechnological minoxidil formulations *in vitro*, *in vivo*, and in clinical studies. Furthermore, an analysis of the total number of selected publications for this study shows that 75% are publications from 2020 onwards, demonstrating their importance in the literature over the last decade. The focus was on physicochemical parameters, comparative efficacy, biocompatibility, and the clinical application feasibility of these agents. In this review, a nanocarrier is defined as a drug delivery system with dimensions smaller than 1000 nm, including polymeric nanoparticles, liposomes, nanoemulsions, nanocrystals, micelles, and lipid nanocarriers (SLN and nanostructured lipid carriers - NLC). This critical synthesis is essential for consolidating existing knowledge and guiding future clinical applications of nanotechnology in the treatment of AGA.

## Methods

### Study criteria

This systematic review was conducted and reported in accordance with the Preferred Reporting Items for Systematic Reviews and Meta-Analyses (PRISMA) 2020 statement and registered in the International Prospective Register of Systematic Reviews (PROSPERO CRD 420251107786).

Two primary reviewers independently extracted the data and compared their selections. Most disagreements were settled through discussions, which often involved revisiting the established study protocol and predefined inclusion/exclusion criteria. If the discussion between the two primary reviewers is insufficient to reach a consensus, a third experienced investigator was consulted to act as an arbitrator and make the final decision.

The PICO criteria, a framework used in evidence-based medicine to formulate focused clinical research questions, were applied in this study: P (Population): adults ≥18-years with AGA, *in vitro* studies (human follicles or skin explants), and *in vivo* mammalian models mimicking AGA; I (Intervention): minoxidil incorporated into nanocarriers (< 1000 nm), including liposomes, SLN, NLC, nano emulsions, and polymeric nanoparticles; C (Comparator): conventional minoxidil (alcoholic solutions or foam), placebo, or absence of intervention; O (Outcomes): primary outcome: follicular and cutaneous penetration. Secondary outcomes included increased hair density, variation in anagen/telogen ratio, local and systemic adverse events, treatment adherence, formulation stability, and clinical feasibility.

The inclusion Criteria included randomized and nonrandomized clinical trials, observational studies, *in vitro* experiments (human follicular cells/skin explants), and *in vivo* mammalian studies published from January 1, 2015, to December 31, 2024, in English, Portuguese, Spanish, French, or Italian.

The exclusion criteria were non-androgenetic alopecias, use of oral minoxidil, non-nanotechnological formulations, absence of therapeutic or physicochemical outcomes, and studies without full text available.

### Information sources

Searches were conducted in PubMed, Embase, Scopus, Web of Science, and ScienceDirect, with the last update scheduled for December 2024. Cross-references from relevant reviews and studies will also be analyzed.

### Search strategy

The search protocol with relevant terms in the title or abstract was as follows: “minoxidil” AND “nanoparticle” OR “nanocarrier” OR “nanoemulsion” OR “nanotechnology” OR “liposome” OR “nanostructured lipid carrier” AND “androgenetic alopecia” OR “AGA”.

The complete strategies adapted for each database are presented in Appendix 1.

### Data extraction

Data will be independently extracted into a standardized spreadsheet, including a) Author/year, country, funding source; b) Nanocarrier type and composition, particle size, and minoxidil concentration; c) Experimental model, number of participants/animals, and treatment duration; d) Clinical, pharmaceutical, and safety outcomes; e) Main results.

### Risk of bias assessment

The risk of bias in animal intervention studies was assessed using the Systematic Review Center for Laboratory Animal Experimentation (SYRCLE) tool, which adapts the Cochrane domains to preclinical *in vivo* experiments.^13^ For toxicological or mechanistic *in vivo* and experiments that did not fit these frameworks, we additionally applied the Office of Health Assessment and Translation (OHAT) of the National Toxicology Program (NTP), a tool that serves as an environmental health resource for the public and regulatory and health agencies.^14^

## Results

Study Selection: A PRISMA flowchart documented this process. After full-text review, two studies were excluded for not containing minoxidil carried in nanoparticles: one evaluated only plant extract without minoxidil, and another study used dutasteride and siRNA in nanoparticles, without including minoxidil in the formulation.

**Figure 1:**
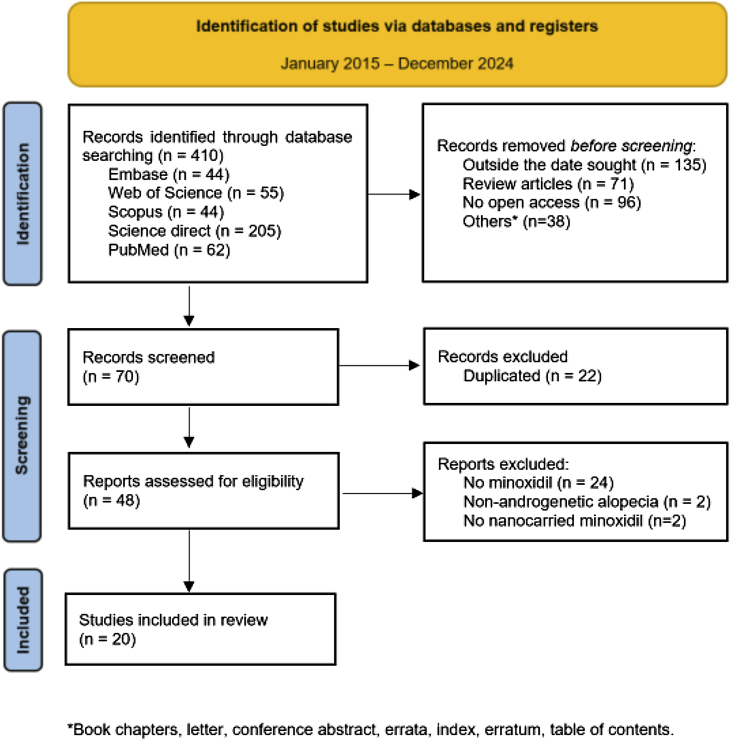
**Nanominoxidil Review: Inclusion Flowchart.** Identification, screening, eligibility and inclusion of studies evaluating nanocarrier-based topical minoxidil for androgenetic alopecia.

The search strategy (Fig. 1) identified 410 records from Embase (n = 44), Web of Science (n = 55), Scopus (n = 44), ScienceDirect (n = 205), and PubMed (n = 62). Of these, 340 records were removed before screening: 135 were outside the predefined date range, 71 were review articles, 96 were not available as open access, and 38 were excluded for being book chapters, letters, conference abstracts, errata, indexes, or tables of contents. The remaining 70 records were subjected to title/abstract screening, during which 22 duplicates were excluded, leaving 48 reports for full-text eligibility assessment. At this stage, 28 reports were excluded: 24 did not evaluate minoxidil as the primary intervention, two did not address androgenetic alopecia, and two did not involve nano-carried minoxidil formulations. Consequently, 20 studies met all the inclusion criteria and were incorporated into the final systematic review, as summarized in Table 1.^15^, ^16^, ^17^, ^18^, ^19^, ^20^, ^21^, ^22^, ^23^, ^24^, ^25^, ^26^, ^27^, ^28^, ^29^, ^30^, ^31^, ^32^, ^33^, ^34^

**Table 1:** Nanotechnology-based minoxidil formulations for the treatment of androgenetic alopecia: Literature Review of Main Characteristics.

First author, Year	Nanocarrier Type	Other Actives	Average Size (nm)	Comparator	Primary Outcomes	Secondary Outcomes	Main Results
Nagai et al., 2019^15^	Solid nanoparticles (bead milling)	NR	153	Microparticles and commercial solution	Deposition in the hair bulb	IGF-1, VEGF	6× ↑ deposition in the bulb vs. microparticles
He et al., 2024^16^	CD-MOF intercross	Cedrol	370	Commercial minoxidil, placebo	Follicular penetration	Hair regeneration, biomarkers	3.06× ↑ follicular retention vs. control; ↑ VEGF and IGF-1
Xiao et al., 2024^17^	Molybdenum nanoparticles	NR	50	Minoxidil 5%, saline	Hair growth	Follicular density, biomarkers	Hair length ↑7.12 mm in combined group
Yan et al., 2023^18^	Ethosomes (MXD + TAT)	Tocopherol acetate	74.3	Commercial minoxidil, physical mixture	Penetration and retention	SULT1A1, Ki67, ROS	4.7× ↑ penetration vs. commercial MXD
Oaku et al., 2022^19^	Solid nanoparticles (bead milling)	NR	139.8	Microparticles and commercial MXD	Deposition in the bulge	IGF-1, VEGF, CD200	7.4× ↑ in the bulge vs. commercial MXD
Oaku et al., 2024^20^	MXD nanocrystals + gum arabic	NR	110.3	Microparticles, 5% commercial lotion	Deposition in the bulge/bulb	CD34, CD200	↑2.6× bulge, ↑3.9× bulb vs. CA-MXD
Li et al., 2023^21^	Ferulic acid-derived lignin NPs	Valproic acid	450	Minoxidil 2%, isolated VPA	Follicular coverage and density	IL-6, ROS, histology	≈4000 hairs/cm^2^, ↓ IL-6 and ROS
Zhang et al., 2024 ^22^	Liposomes + exosomes in hydrogel	NR	171.2	Minoxidil 5%, empty hydrogel	Hair coverage	VEGF, CD31, transcriptomics	↑ density and VEGF vs MXD 5%
Xing et al., 2024^23^	Hyaluronic acid liposomes + NO	NO donor	200	Commercial minoxidil, liposomes without NO	Cutaneous penetration	Angiogenesis, biomarkers	↑ penetration, ↑ VEGF, ↓ IL-6
Ramezani et al., 2018^24^	Transfersomes (MXD + caffeine)	Caffeine	NR	Aqueous and hydroalcoholic solution	Hair growth	Controlled release	↑ hair length and weight vs. solution
Kochar et al., 2019^25^	Liposomes (MXD + TRET) in hydrogel	Tretinoin	149.3	Mintop™ and Nioret™	Cutaneous permeation and retention	Stability, irritation	↑ dermal retention of TRET and MXD without irritation
Oliveira et al., 2022^26^	NLC (MXD + Latanoprost)	Latanoprost	393.3	Aqueous MXD + LAT solution	Follicular penetration	Cell viability, VEGF	2× ↑ follicular deposition
Fresta et al., 2020^27^	Liquid crystalline nanocarriers	NR	82	Minoxidil 5%, SLNs, transfersomes	Follicular penetration	Hair growth, tolerability	↑ retention 91% vs. 13% solution
Aljuffali et al., 2015^28^	Squarticles with PDGF-β antibody	NR	194.5	MXD 20% PG solution	Follicular deposition and DPC internalization	VEGF, cell proliferation	8.7× ↑ cutaneous deposition vs. solution
Sun et al., 2023^29^	Transfersomes + gel	NA	76.7	Commercial minoxidil	Cutaneous permeation	Hair growth, histology	↑ penetration and deposition vs. control
Makhlouf et al., 2023^30^	Cubosomes	NR	131.1	0.5% alcoholic solution	Hair growth	Cutaneous penetration, histology	↑ penetration and growth vs. solution
Melo-Guímaro et al., 2024^31^	MNX@β-CD complex + photoacoustic waves	NR	NR	Minoxidil 2% commercial	Transdermal flux	Hair coverage, blood pressure	↑ active penetration 3× vs. passive; 86% coverage
Jeong et al., 2019^32^	PLGA-HA NPs	NR	243	Aqueous MXD solution	Transdermal penetration	Cell uptake, viability	↑ cutaneous penetration (150.6 μg) vs. PLGA without HA
Liu et al., 2024^33^	Transfersomes + ginsenoside Rg3	Rg3	84.2	Conventional transfersomes, MXD 2%	Hair growth	Inflammation (IL-6, TNF-α), DHT	↑ hair length/diameter, ↓ DHT
Al-Taie et al., 2024^34^	Nanoemulsion/Nanoemulgel	NR	14.6	Hairgrow® 2%, 1% solution	Cutaneous permeation	Release, stability	2.3× ↑ permeation vs. Hairgrow®

Summary of the nanocarrier-based topical minoxidil formulations evaluated in the included studies on androgenetic alopecia.

NR, Not Reported; MXD, Minoxidil; CDF, Cyclodextrin Metal-Organic Framework; hDPCs, human Dermal Papilla Cells; HUVECs, Human Umbilical Vein Endothelial Cells; DPCs, Dermal Papilla Cells; VEGF, Vascular Endothelial Growth Factor; IGF-1, Insulin-Like Growth Factor-1; TGF-β, Transforming Growth Factor Beta; PCNA, Proliferating Cell Nuclear Antigen; ROS, Reactive Oxygen Species; SULT1A1, Sulfotransferase-1A1; Jss, Steady-state permeation flux; DR, Skin Drug Deposition; RNA-seq, RNA sequencing; IL-6, Interleukin-6; TNF-α, Tumor Necrosis Factor-alpha; DHT, Dihydrotestosterone; CD31, Endothelial marker; CD34/CD200, Hair follicle stem cell markers; ECM, Extracellular Matrix; MOF, metal organic frame work; VPA, valproic acid; TAT, tocopherol acetate; NPs, nanoparticules; NO, nitric oxide; TRET. tretinoin; LAT, latanoprost; SLN, solid lipid nonoparticles; MNX@beta-CD, minoxidil partially encapsulated in β-cyclodextrin; PLGA-HA, polylactic-co-glycolic acid and hydroxyapatite.

Table 1 presents the major characteristics, general and specific outcomes, and descriptions of the study methodologies and tools employed, including the unique study number (from 1 to 20), authors and year of publication, type of nanocarrier utilized, any other active ingredients included in the formulation, average particle size in nanometers, comparators employed in the study, experimental model used, primary outcomes investigated, secondary outcomes assessed, and main results reported. Table 1 provides a detailed overview of each of the included studies.^15^, ^16^, ^17^, ^18^, ^19^, ^20^, ^21^, ^22^, ^23^, ^24^, ^25^, ^26^, ^27^, ^28^, ^29^, ^30^, ^31^, ^32^, ^33^, ^34^

Most studies have been conducted in animals or *in vitro*, indicating a lack of nanominoxidil research in humans. Follicular penetration of nanominoxidil was the primary outcome of the major studies, measured in μg/cm^2^. It is relevant to note that the authors cite different types of methods for quantifying the drug in hair follicles, using various techniques (e.g., SEM - scanning electron microsocpy, FTIR - Fourier transform infrared spectroscopy, PXRD - Powder X-ray diffraction), and confocal laser scanning microscopy), which lead to direct interference in statistical results.

Nagai et al.^15^ examined solid minoxidil nanoparticles in mice using molecular analysis and evaluated their deposition in the hair bulb and hair growth. The levels of insulin-like growth factor-1 (IGF-1) and vascular endothelial growth factor (VEGF) were higher in the presence of minoxidil nanoparticles than with minoxidil, according to the authors’ analysis. They also confirmed higher levels of minoxidil nanoparticles in the target tissue than regular minoxidil, concluding that nanotechnology minoxidil would be more effective and as safe as the traditional formulation.

Most studies performed biomarker analyses. The biomarkers include VEGF, IGF-1, β-catenin, Ki67, reactive oxygen species (ROS), sulfotransferase family 1A member 1 (SULT1A1), CD200, IL-6, CD31. Objective and statistically relevant quantifications are **e**ssential to ensure replicable safety and efficacy of drug results.^15^, ^16^, ^17^, ^18^, ^19^, ^20^, ^21^, ^22^

Skin irritation is a common clinical outcome of topical minoxidil use. It can be manifested by redness, itching, and/or scales in the scalp skin, which is the reason for suspending the use of topical minoxidil. Skin penetration was the most prevalent primary outcome reported in the reviewed studies, as presented in Table 1. There is an explicit lack of standardization in the reviewed studies, even for naming outcomes. We contemplate that 50% of the studies report “skin penetration” as the primary outcome when considering terms “cutaneous penetration or permeation”, “transdermal penetration”, “bulge or bulb penetration or deposition” and “follicular penetration” as the same as “skin penetration” outcome.

Nanotechnology also offers the benefit of delivering other active ingredients in conjunction with minoxidil. In this vein, Xing et al.^23^ presented the co-delivery of vasodilator nitric oxide with minoxidil via functionalized liposomes. The authors evaluated vessel growth (neoangiogenesis) and the stability of the formulation. Similarly, Yan et al.^18^ explored the co-delivery of MXD and tocopherol acetate using a testosterone-induced AGA model. Ramezani et al.^24^ investigated transferosomes containing MXD and caffeine. In this context, Kochar et al.^25^ focused on the co-delivery of MXD and tretinoin in liposomes and evaluated their cutaneous permeation and retention. Li et al.^21^ utilized lignin nanoparticles (NPs) with valproic zcid (VPA) associated with microneedling in a murine AGA model.

Oliveira et al.^26^ investigated the development of NLCs for the topical co-delivery of MXD and latanoprost in androgenetic alopecia. This nanotechnology aims to overcome the limitations of conventional solutions, such as suboptimal skin penetration and potential systemic effects of topical formulations. This study compared NLC-based formulations (MXD 5% combined with latanoprost at 0.005% and 0.010%) with non-nanostructured topical solutions. NLCs demonstrated significantly superior performance, achieving up to a two-fold enhancement in follicular deposition (Table 1 of the systematic review) and reduced systemic permeation. This underscores the advantages of nanotechnology in targeted drug delivery to hair follicles, maximizing local therapeutic effects and improving safety.

To compare different nanominoxidil formulations, the group led by Fresta et al.^27^ conducted a comprehensive study using *in vitro* and *in vivo* models and human volunteers. The formulations compared were SLNs, transferosomes, and hydroalcoholic solutions, followed by hair growth assessment.

Sustained drug release is another well-known advantage of nanotechnology over regular formulations. Aljuffali et al.^28^ developed squarticles conjugated to a platelets-derived growth factor-beta (PDGF-β) antibody, evaluating cutaneous penetration and internalization in dermal papilla cells (DPCs), and demonstrating sustained release of nanominoxidil in this target.

Table 2^15^, ^16^, ^17^, ^18^, ^19^, ^20^, ^21^, ^22^, ^23^, ^24^, ^25^, ^26^, ^27^, ^28^, ^29^, ^30^, ^31^, ^32^, ^33^, ^34^ presents the results of the included studies stratified by their experimental model (*in vitro/ex vivo, in vivo*, and clinical). Some studies are listed more than once, as they reported results from different experimental stages, frequently assessing distinct parameters across *in vitro* and *in vivo* models within the same investigation. This stratification facilitates the interpretation of heterogeneous study designs and outcome measures. However, a substantial variability in experimental duration was observed, ranging from hours to several weeks, depending on the model and endpoint evaluated. This wide disparity in study duration, combined with differences in assessed parameters, limits direct comparability between studies and complicates the interpretation of the temporal dynamics and sustained effects of nanominoxidil formulations.

**Table 2:** Stratification, model, assessed parameters and type of co-adjuvant substance of topical nanominoxidil for hair disorders: literature review.

Category	Author/Year	Model	Assessed Parameters	Study duration
*In vitro/Ex vivo*	He et al., 2024^16^	hDPCs + pig skin	Follicular penetration, VEGF, IGF-1, TGF-β, cytotoxicity	21 days
	Yan et al., 2023^18^	L929 + hDPC + pig skin	Cutaneous/follicular penetration, Ki67, ROS, SULT1A1	24 days
	Li et al., 2023^21^	L929 cells	Cytotoxicity, cumulative release, ROS	12 days
	Zhang et al., 2024^22^	L929 + Raw 264.7	Cytotoxicity, transcriptomics	13 days
	Xing et al., 2024^23^	HUVECs + hDPCs	Cutaneous penetration, Ki67, PCNA, VEGF, β-catenin, IL-6, TGF-β	21 days
	Kochar et al., 2019^25^	Rat skin	MXD and TRET permeation, cutaneous retention	3 days
	Oliveira et al., 2022^26^	Human keratinocytes + pig skin	Follicular penetration, MKI67, VEGF, cell migration	24 hours
	Fresta et al., 2020^27^	Human keratinocytes + human SCE membranes	Follicular penetration and cutaneous distribution	28 days
	Aljuffali et al., 2015^28^	Pig skin + human DPCs	Follicular deposition, VEGF, DPC internalization	4 hours
	Sun et al., 2023^29^	Pig and guinea pig skin	Cutaneous permeation (Jss, μg/cm²/h), dermal deposition	24 hours
	Makhlouf et al., 2023^30^	*In vitro* release	Release kinetics, confocal penetration	21 days
	Melo-Guímaro et al., 2024^31^	Pig skin	Transdermal flux, layer deposition	28 days
	Jeong et al., 2019^32^	NIH-3T3 fibroblasts + rat skin	Transdermal penetration, cell uptake, viability	10 days
	Liu et al., 2024^33^	Human DPCs	Cell viability, IL-6, TNF-α, ROS	7 days
	Al-Taie et al., 2024^34^	Rat skin	Cutaneous permeation, cumulative release, stability	21 days
In vivo	Nagai et al., 2019^15^	C57BL/6 mice	Bulb/bulge deposition, IGF-1, VEGF	15 days
	He et al., 2024^16^	C57BL/6 mice	Regenerated hair weight, anagen/telogen ratio, histology	21 days
	Xiao et al., 2024^17^	C57BL/6 mice	Hair length and density, CD34+, iNOS, COX-2, RNA-seq	16 days
	Yan et al., 2023^18^	C57BL/6 mice	Hair coverage and length, histology	24 days
	Oaku et al., 2022^19^	C57BL/6 mice	Hair growth area, IGF-1, VEGF, CD200	16 days
	Oaku et al., 2024^20^	C57BL/6 mice	Cutaneous distribution (bulge/bulb), CD34, CD200	12 days
	Li et al., 2023^21^	C57BL/6 mice	Hair density/coverage, IL-6, ROS, histology	12 days
	Zhang et al., 2024^22^	C57BL/6 mice	Hair coverage/density, CD31, VEGF	13 days
	Xing et al., 2024^23^	C57BL/6 mice	Coverage area, hair weight, histology	21 days
	Ramezani et al., 2018^24^	Wistar rats	Hair length and weight	30 days
	Fresta et al., 2020^27^	Wistar rats	Hair length, % growth in alopecia	28 days
	Aljuffali et al., 2015^28^	Nude mice	Cutaneous distribution and penetration	4 hours
	Sun et al., 2023^29^	C57BL/6 mice	Hair length and histology	24 hours
	Makhlouf et al., 2023^30^	Wistar rats	Hair growth scale (0–5), histology	21 days
	Melo-Guímaro et al., 2024^31^	Sprague-Dawley rats	Hair coverage, blood pressure	28 days
	Liu et al., 2024^33^	C57BL/6 mice	Hair length/diameter, DHT, IL-6, TNF-α	7 days
Clinic	Fresta et al., 2020^27^	Healthy volunteers	Cutaneous tolerability, erythema	28 days

Summary of the included studies grouped by experimental category, detailing the biological model, main pharmacokinetic and pharmacodynamic parameters and the type of coadjuvant substance in topical nanominoxidil formulations.

MXD, Minoxidil; CDF, Cyclodextrin Metal-Organic Framework; hDPCs, Human Dermal Papilla Cells; HUVECs, Human Umbilical Vein Endothelial Cells; DPCs, Dermal Papilla Cells; VEGF, Vascular Endothelial Growth Factor; IGF-1, Insulin-like Growth Factor-1; TGF-β, Transforming Growth Factor Beta; PCNA, Proliferating Cell Nuclear Antigen; ROS, Reactive Oxygen Species; SULT1A1, Sulfotransferase-1A1; Jss, Steady-State permeation flux; DR, Skin Drug Deposition; RNA-seq: RNA sequencing; IL-6, Interleukin-6; TNF-α, Tumor Necrosis Factor-alpha; DHT, Dihydrotestosterone; CD31, Endothelial marker; CD34/CD200, Hair follicle stem cell markers; ECM, Extracellular Matrix; L929, standard murine fibroblast cell line; TRET, tretinoin; VEGF, vascular endothelial growth factor; IGF-1, insulin-like growth factor 1; SCE, selective layer-composite; iNOS, inducible nitric oxide synthase; COX-2, cyclooxygenase-2; NIH-3T3, mouse embryonic fibroblast cell line.

Statistical comparison of enhanced minoxidil formulations over isolated minoxidil or control, based on primary outcomes reported in the included studies evaluating nanocarriers and presented as the mean difference between nanominoxidil and the respective control (conventional minoxidil, placebo, or comparator formulation), is provided in Table 3.^15^, ^16^, ^17^, ^18^, ^19^, ^20^, ^21^, ^22^, ^23^, ^24^, ^25^, ^26^, ^27^, ^28^, ^29^, ^30^, ^31^, ^32^, ^33^, ^34^ For each outcome, the self-reported p-value is presented to facilitate the assessment of statistical significance. This table visually illustrates instances in which enhanced minoxidil formulations demonstrated superior efficacy compared with isolated minoxidil or control interventions, highlighting their potential advantages in the treatment of androgenetic alopecia. Appendix 1 presents the complete data of the articles revised in this paper, including the risk of bias applied to our research, which used SYRCLE and OHAT tools, as described in the methods.

**Table 3:** Statistical comparison of enhanced minoxidil formulations over isolated minoxidil or control.

Author, year	Primary outcome	Mean Difference and Control (95% CI)	p-value
Nagai et al., 2019^15^	Deposition in bulb (μg/g)	6× ↑ vs. microparticles (95% CI 5.2–6.8)	<0.001
He et al., 2024^16^	MXD follicular penetration (μg/cm^2^)	3.06× ↑ vs. crude MXD (95% CI 2.7–3.4)	<0.01
Xiao et al., 2024^17^	Hair length (mm)	+2.1 mm vs. NaCl (95% CI 1.8–2.4)	<0.001
Yan et al., 2023^18^	MXD follicular penetration (μg/cm^2^)	4.7× ↑ vs. commercial MXD (95% CI 4.1–5.3)	<0.001
Oaku et al., 2022^19^	Concentration in the bulge (μg/g)	7.4× ↑ vs. commercial MXD (95% CI 6.5–8.3)	<0.001
Oaku et al., 2024^20^	Deposition in bulge (μg/g)	2.6× ↑ vs. CA-MXD (95% CI 2.2–3.0)	<0.001
Li et al., 2023^21^	Hair density (hairs/cm^2^)	+2000 hairs/cm^2^ vs. control (95% CI 1800–2200)	<0.001
Zhang et al., 2024^22^	Hair coverage (%)	+24% vs. MXD 5% (95% CI 18–30%)	<0.001
Xing et al., 2024^23^	Hair coverage area (%)	+38% vs. commercial MXD (95% CI 32–44%)	<0.001
Ramezani et al., 2018^24^	Hair length (mm)	+1.5 mm vs. aqueous solution (95% CI 1.2–1.8)	<0.01
Kochar et al., 2019^25^	TRET cutaneous retention (%)	+14.4% vs. simple liposomes (95% CI 11–18%)	<0.05
Oliveira et al., 2022^26^	LAT follicular penetration (μg/cm^2^)	3× ↑ vs. solution (95% CI 2.6–3.4)	<0.01
Fresta et al., 2020^27^	Follicular retention (%)	91% vs. 13% (95% CI 85–97%)	<0.001
Aljuffali et al., 2015^28^	Follicular deposition (μg/cm^2^)	8.7× ↑ vs. PG solution (95% CI 7.5–9.9)	<0.001
Sun et al., 2023^29^	Cutaneous deposition (μg/cm^2^)	+96.7 μg/cm^2^ vs. control (95% CI 88–105)	<0.001
Makhlouf et al., 2023^30^	Hair growth score (0–5)	+0.3 vs. MXD solution (95% CI 0.1–0.5)	<0.05
Melo-Guímaro et al., 2024^31^	Hair coverage (%)	+32% vs. MXD 2% (95% CI 26–38%)	<0.001
Jeong et al., 2019^32^	Cutaneous penetration (μg)	+34.9 μg vs. PLGA without HA (95% CI 30–39)	<0.01
Liu et al., 2024^33^	Hair diameter (μm)	+5.76 μm vs. commercial MXD (95% CI 5.1–6.4)	<0.001
Al-Taie et al., 2024^34^	Cumulative cutaneous permeation (μg/cm^2^)	2.3× ↑ vs. Hairgrow® (95% CI 2.1–2.5)	<0.001

Primary outcomes reported in the included studies evaluating nanocarrier formulations of topical minoxidil for androgenetic alopecia, presented as the mean difference between nanominoxidil and the respective control (conventional minoxidil, placebo or comparator formulation), with corresponding 95% Confidence Interval (95% CI) and p-value as reported by the original authors. Positive values or fold-changes indicate superiority of the nanocarrier formulation over its control.

MXD, Minoxidil; LAT, Latanoprost; TRET, Tretinoin; CA-MXD, Conventional Alcoholic Minoxidil; PG, Propylene Glycol; HA, Hyaluronic Acid; NLC, Nanostructured Lipid Carrier; PLGA, Polylactic-co-Glycolic Acid.

## Discussion

The goal of most revised studies was to evaluate the capacity for skin penetration or accumulation after the active delivery of minoxidil to the desired target, in the case of androgenetic alopecia, the hair follicle, specifically the follicular bulge. This optimization of targeted delivery is clearly the main interest in the scenario of nanotechnology benefits.

The methodological variation among studies is a concern for this review. The absence of a randomized analysis in the articles is one of the most important points to present as a weakness in the literature and perspectives for the future. Huge variation in the study duration is another subjective scenario, where some do not describe it specifically, some present hours, and some others present a 30-day stability study.

A significant limitation identified in the reviewed literature is the lack of standardization in study parameters and outcome measures, which profoundly hinders direct comparisons between studies. For instance, while some studies prioritize follicular penetration, others focus on hair length, cutaneous deposition, and follicular retention. This heterogeneity in primary outcomes makes it challenging to ascertain which formulation truly offers superior benefits, and prevented this review from conducting a meta-analysis due to non-comparable data. Furthermore, the duration of these studies is often inconsistent and non-standardized; some evaluate the impact of formulations within hours, others after days, and many lack analyses at multiple time points, as presented in the “study duration” section at Table 2. This variability further complicates the direct comparison of results. Consequently, there is an urgent need for standardization in the design and execution of studies involving minoxidil-loaded carriers and nanocarriers. Such uniformity is essential to understand the comparative impact of different nanocarrier systems and advance the field towards more conclusive evidence.

In several studies, nanominoxidil was not used as the sole intervention, which raises the question of how these factors can influence outcomes. Table 1 presents “other actives” section to comparison of the reviewed studies. It is important to highlight that different adjuvant agents, when combined with nanominoxidil, may act synergistically to achieve different outcomes. Examples of recently studied compounds associated with minoxidil include tretinoin, finasteride, corticoids, exosomes, and growth factors. Tretinoin may reduce the thickness of the stratum corneum and increase skin permeability, allowing minoxidil to penetrate more deeply. The minoxidil-tretinoin combination, especially in advanced nano delivery systems such as liposomes, increases the number of anagen hairs and hair density, which might be useful even for patients who do not respond to minoxidil alone.^35^ The combination of minoxidil with finasteride (a 5α-reductase inhibitor) in nanoparticles (such as hybrid vesicles) is believed to allow higher *in situ* delivery of the 5α-reductase inhibitor.^36^ The use of mild corticosteroids as adjuncts in propylene glycol-free vehicles might reduce itching and dermatitis, which could increase adherence to treatment (over 80% adherence vs. ∼45% for commercial minoxidil), which can directly result in better long-term clinical outcomes.^37^

Agents such as stem cell-derived exosomes or Wnt/β-catenin pathway stimulators can be co-encapsulated with minoxidil, and further studies must be conducted to confirm whether it is possible to promote the activation of hair follicle stem cells more rapidly than nanominoxidil alone, as well as the viability of proper use of exosomes outside a laboratory environment.^38^

To this end, we propose the establishment of minimum mandatory parameters for all future studies, enabling comparability and facilitating subsequent analysis. For *in vitro* and *ex-vivo* studies, follicular penetration and retention after 24 hours are crucial. In *in vivo* human clinical trials, metrics such as hair density (hairs/cm^2^) and hair diameter (in micrometers) should be consistently assessed at baseline, after 30-days, and after 6-months (or 24-weeks). Table 1 presents different “nanocarriers type” and different “coadjuvant substances” among reviewed studies, which brings the discerning that comparisons with similar nanocarriers and those without the weakness of coadjuvant substances are important avenues for future research.

Beyond outcome variability, many of the reviewed studies suffer from inherent methodological limitations. Most studies have been conducted in animal models, revealing a significant gap in human scientific evidence and highlighting the need for future studies in humans. Common shortcomings include short study durations, absence of blinding, insufficient descriptions of other scientific rigor tools, scarce data on adverse events, and, in some cases, disclosed conflicts of interest with pharmaceutical companies. All these aspects represent significant scientific limitations, which are further exemplified by the individual appraisals of the included studies.

An individual appraisal revealed that while He et al.^16^ demonstrated comprehensive physicochemical characterization and a multi-model approach with biomarker analysis, a notable limitation was the absence of clinical trials and unclear reporting on randomization and blinding. Similarly, Xiao et al.,^17^ despite using an *in vivo* model with extensive molecular analyses, had a short study duration, a lack of detail on randomization and blinding, and failure to report adverse events. Xing et al.^23^ showed strengths in co-delivery and molecular analysis, but lacked clinical trials, failed to describe randomization or blinding, and reported only the short-term stability of the formulation. Al-Taie et al.^34^ provided extensive physicochemical characterization and stability data, but its limitations included being restricted to *in vitro* and *ex vivo* studies, lacking *in vivo* efficacy assessment, and not reporting adverse events.

Yan et al.^18^ explored co-delivery in an AGA model with biomarker and stability assessment, yet key limitations were a short follow-up period, absence of blinding or randomization, and no human testing. Sun et al.^29^ developed a robust formulation with detailed histology and stability; however, the absence of clinical trials and undocumented randomization or blinding were crucial shortcomings. Makhlouf et al.^30^ introduced an innovative cubosome formulation with detailed penetration and hair growth assessment, but did not include clinical trials and omitted reporting randomization and blinding. Oaku et al.^19^ focused on targeted nanoparticles with molecular analysis and confirmed no systemic detection of MXD; however, their study had limitations, including the lack of clinical trials, a restricted stability period, and a declared conflict of interest.

Oliveira et al.^26^ investigated NLCs for co-delivery, performing extensive *ex vivo* and *in vitro* evaluations; however, the absence of *in vivo* and clinical trials, along with unreported blinding or randomization, made it challenging to extrapolate these findings to humans. Fresta et al.^27^ offered a comprehensive study including human volunteers, yet its limitations included a lower MXD dose than standard concentrations, a small sample size in the human study, and the absence of a controlled clinical trial. Kochar et al.^25^ focused on co-delivery in liposomes with permeation and retention assessment and stability data, but the lack of *in vivo* efficacy studies and clinical trials limited the understanding of its actual therapeutic potential. Nagai et al.^15^ examined solid nanoparticles using molecular analysis and deposition evaluation; however, similar to Oaku et al.,^19^ their limitations included the absence of clinical trials and a declared conflict of interest.

Ramezani et al.^24^ investigated transferosomes with *in vivo* hair growth, controlled release, and stability assessment; however, incomplete physicochemical parameters and the absence of clinical trials affected reproducibility and clinical relevance. Aljuffali et al.^28^ developed articles with detailed penetration and internalization studies, but the primary limitations were the lack of hair growth assessment and clinical trials. Melo-Guímaro et al.^31^ explored a complex combined with photoacoustic waves, using i*n vivo* and *ex vivo* models, but its limitations included the absence of a specific AGA model, no long-term effect evaluation, and a declared conflict of interest. Li et al.^21^ utilized lignin nanoparticles in a murine AGA model, including histological and inflammatory analyses; however, their shortcomings included a short follow-up period and the absence of clinical trials.

Zhang et al.^22^ developed a hydrogel with liposomes and exosomes, performing transcriptomic and angiogenesis assessment, yet the lack of clinical trials and long-term stability data hindered the assessment of its real-world application. Jeong et al.^32^ focused on polylactic-co-glycolic acid and hydroxyapatite (PLGA-HA) nanoparticles, evaluating permeation, cellular uptake, and prolonged release; however, the absence of *in vivo* hair growth tests and clinical trials was a significant limitation. Oaku et al.^20^ developed nanocrystals demonstrating increased follicular delivery and reduced systemic absorption; however, limitations included the lack of clinical trials and a declared conflict of interest. Finally, Liu et al.^33^ presented modified transferosomes with synergistic effects and increased hair parameters; however, the absence of clinical trials and long-term evaluation made the sustainability of these effects unclear.

Despite these methodological gaps, some studies have demonstrated important strengths in this area. Many studies have excelled in the detailed physicochemical characterization of nanoparticles and their carriers, and the use of multi-model approaches (*in vitro*, *ex vivo*, and *in vivo*) provided valuable insights. Analysis of biomarkers (e.g., VEGF, IGF-1, β-catenin) and *in vitro* angiogenesis assays further corroborate minoxidil's potential. Fresta et al.^27^ analysis of cutaneous tolerability in human tissue, though not a clinical trial, suggests nanominoxidil superiority in follicular deposition and absence of erythema. However, the imprecise description of skin irritation reported by He et al.^16^ highlights the need for more detailed clinical evaluations, including specific signs and symptoms. The co-evaluation of nanominoxidil with other active ingredients, as seen by Oliveira et al.^26^ with latanoprost, which emphasizes the promising potential of lipid nanocarrier systems for targeted minoxidil delivery and the need for absorption, distribution, metabolism and excretion (ADME) studies.

Randomized, controlled, and comparative clinical trials involving minoxidil nanoparticles in human subjects, utilizing tools such as blinding and prolonged study durations (e.g., 24-weeks), represent areas of interest in scientific literature. Standardization of parameters, especially the proposed minimums (duration, drug concentration, and comparison of the same objective outcome), will be fundamental for the advancement and comparability of these studies.

## Conclusion

The application of nanotechnology to minoxidil for hair growth across various pathologies, including androgenetic alopecia, offers significant potential for future therapeutic applications. To date, no randomized clinical trials have compared the efficacy of minoxidil nanoparticles with that of conventional topical minoxidil and/or low-dose oral minoxidil for the treatment of androgenetic alopecia. We highlight the urgent need for rigorous, standardized future research describing the efficacy and safety of nanominoxidil for hair disorders in humans.

## ORCID ID

Gustavo Sartori Albertino: 0000-0003-0728-7828

Filipe Rocha Lima: 0000-0001-7629-4963

Antonio Claudio Tedesco: 0000-0003-4198-9321

Marco Andrey Cipriani Frade: 0000-0003-2700-5971

## Ethical considerations

As this study is a systematic review of previously published data, approval by a Research Ethics Committee and informed consent from participants were not required. The protocol was prospectively registered in the International Prospective Register of Systematic Reviews (PROSPERO number CRD420251107786).

## Research data availability

The entire dataset supporting the results of this study was published in this article.

## Financial support

This study was financed in part by Coordenação de Aperfeiçoamento de Pessoal de Nível Superior – Brazil (CAPES) [Finance Code 001]. The funding agency had no role in study design, data collection, data analysis, manuscript preparation, or the decision to submit the article for publication.

## Authors’ contributions

Heloisa da Rocha Picado Copesco: The study concept and design; data collection, or analysis and interpretation of data; statistical analysis; writing of the manuscript or critical review of important intellectual content; intellectual participation in the propaedeutic and/or therapeutic conduct of the studied cases; critical review of the literature; final approval of the final version of the manuscript.

Gustavo Sartori Albertino: The study concept and design; data collection, or analysis and interpretation of data; statistical analysis; writing of the manuscript or critical review of important intellectual content; intellectual participation in the propaedeutic and/or therapeutic conduct of the studied cases; critical review of the literature; final approval of the final version of the manuscript.

Filipe Rocha Lima: Critical review of important intellectual content; critical review of the literature; final approval of the final version of the manuscript.

Antonio Claudio Tedesco: Critical review of important intellectual content; critical review of the literature; final approval of the final version of the manuscript.

Marco Andrey Cipriani Frade: Critical review of important intellectual content; critical review of the literature; final approval of the final version of the manuscript.

## Conflicts of interest

The authors declare that the research was conducted in the absence of any commercial or financial relationships that could be construed as a potential conflict of interest.
